# Neutrophil-dependent Mitochondrial DNA Release Associated With Extracellular Trap Formation in Inflammatory Bowel Disease

**DOI:** 10.1016/j.gastha.2023.03.022

**Published:** 2023-03-23

**Authors:** Broc Drury, Cher S. Chuah, Rebecca Hall, Gareth R. Hardisty, Adriano G. Rossi, Gwo-Tzer Ho

**Affiliations:** Edinburgh IBD Science Unit, Centre for Inflammation Research, University of Edinburgh, Edinburgh, UK

**Keywords:** IBD, UC, CD, NETosis, Mitochondria, cfDNA

## Abstract

**Background and Aims:**

Inflammatory bowel disease (IBD) is associated with increased circulating damage-associated molecular patterns, in particular, the highly pro-inflammatory mitochondrial DNA (mtDNA). Here, we study the importance of blood neutrophils in mtDNA release via neutrophil extracellular trap (NET) formation and mitochondrial NETosis, where neutrophils specifically expulse mtDNA as potential targetable biological pathways.

**Methods:**

We investigated the roles of A23187 (a known NET stimulant), granulocyte macrophage stimulating factor, lipopolysaccharide (LPS), and human IBD plasma in their ability to induce NET formation, mitochondrial NETosis, mtDNA, and total DNA release from human blood neutrophils; and the evidence for increased NET formation in IBD.

**Results:**

We demonstrated that NET formation resulted in significant DNA (*P* < .0001) and mtDNA release (*P* < .0001) with long DNA fragments (>1000 base pairs) with NETs containing high levels of mtDNA. Using previously described in vitro conditions for mitochondrial NETosis, granulocyte macrophage stimulating factor + LPS triggered neutrophil mtDNA release at lower levels but not NETosis. LPS alone can trigger neutrophilic DNA release without NET formation. Heterologous coculture with plasma from patients with active IBD (vs remission [n = 6/group]) were not associated with significantly higher levels of NETs and mtDNA release. During coculture with active IBD plasma (vs remission), citrullinated histone 3 (CitH3) (a NETs biomarker) levels were significantly lower (*P* < .001). Similarly, CitH3 levels were lower in stool supernatants of patients with active IBD vs remission (n = 19/12, *P* = .0001). Stool CitH3 negatively correlates with stool calprotectin, a biomarker for gut inflammation (r = −0.47, *P* = .03).

**Conclusion:**

Hence, although blood neutrophils remain an important source of circulating mtDNA with defined mechanisms for release via NET formation and during neutrophil activation, our data do not support excessive systemic NET formation as a dominant underpinning pathobiological process in IBD.

## Introduction

Inflammatory bowel diseases (IBDs), ulcerative colitis (UC), and Crohn’s disease (CD) are common immune-mediated conditions affecting the gastrointestinal tract. Although UC and CD have many different clinical manifestations, they share a fundamental characteristic: an abnormal gut inflammatory process that fails to resolve. The most widely accepted general hypothesis for IBD is that genetic susceptibility and environmental factors contribute to perturb the gut mucosal barrier, altering the healthy balance of the gut microbiota, and abnormally stimulating gut immune response leading to the development of chronic inflammation and longer-term gut tissue damage. Hence, pathogenic factors contributing to the perpetuation of chronic inflammation represent potential targets for therapeutic intervention.

We (and others) recently demonstrated high circulating mitochondrial DNA levels in the blood and stools of patients with IBD, which are associated with clinical and endoscopic markers of gut inflammation.^1^^,^^2^ Mitochondria are descended from bacteria and retain many of their ancestor’s properties. Most notably, mitochondrial DNA possesses unmethylated CpG motifs similar to bacterial DNA that can act as a damage-associated molecular pattern to trigger the innate immune system.^3^ Hence, high mitochondrial DNA levels in active IBD present an interesting scenario in line with other conditions where liberated mitochondrial cfDNA becomes a trigger for inflammation, in a spectrum of conditions from various forms of sepsis to chronic immune-mediated diseases such as systemic lupus erythematosus.^4^^,^^5^

A key question consequent to this observation is the nature of the biological process leading to mitochondrial DNA release in IBD. Current data show that host cfDNA (including genomic) originates mostly from hematopoietic cell death.^6^ In health, Moss et al. estimate that circulating cell-free DNA is derived from 55% white blood cells, 30% erythrocyte progenitors, and 10% vascular endothelial cells, accounting for ∼95% of cfDNA.^6^ In 2004, Brinkmann *et al.* vividly described the expulsion of long sticky DNA strands, and histones laced with an array of granule proteins by neutrophils when activated by interleukin 8, phorbol 12-myristate 13-acetate, or lipopolysaccharide (LPS) as a defense mechanism: the neutrophil extracellular trap (NET).^7^ NET formation is important in gut mucosal host defense particularly against fungal infections. Several lines of evidence suggest that excessive NET formation is present in IBD and may contribute to the perpetuation of the abnormal gut inflammatory response.^8^ In our study, we specifically investigate the role of NET formation in mitochondrial DNA release in IBD. We also evaluated the potential mechanism of mitochondrial NETs—a defined process where neutrophils in certain conditions undergo NETosis that specifically releases mitochondria DNA as the main constituents of the extracellular trap.^9^ Secondly, we sought evidence for NETosis in active IBD, as a relevant process for higher mitochondrial DNA release in IBD.

## Results

### NET formation in human peripheral blood neutrophils and SYTOX Green neutrophil-labeling assay for NETosis

**Figure 1:**
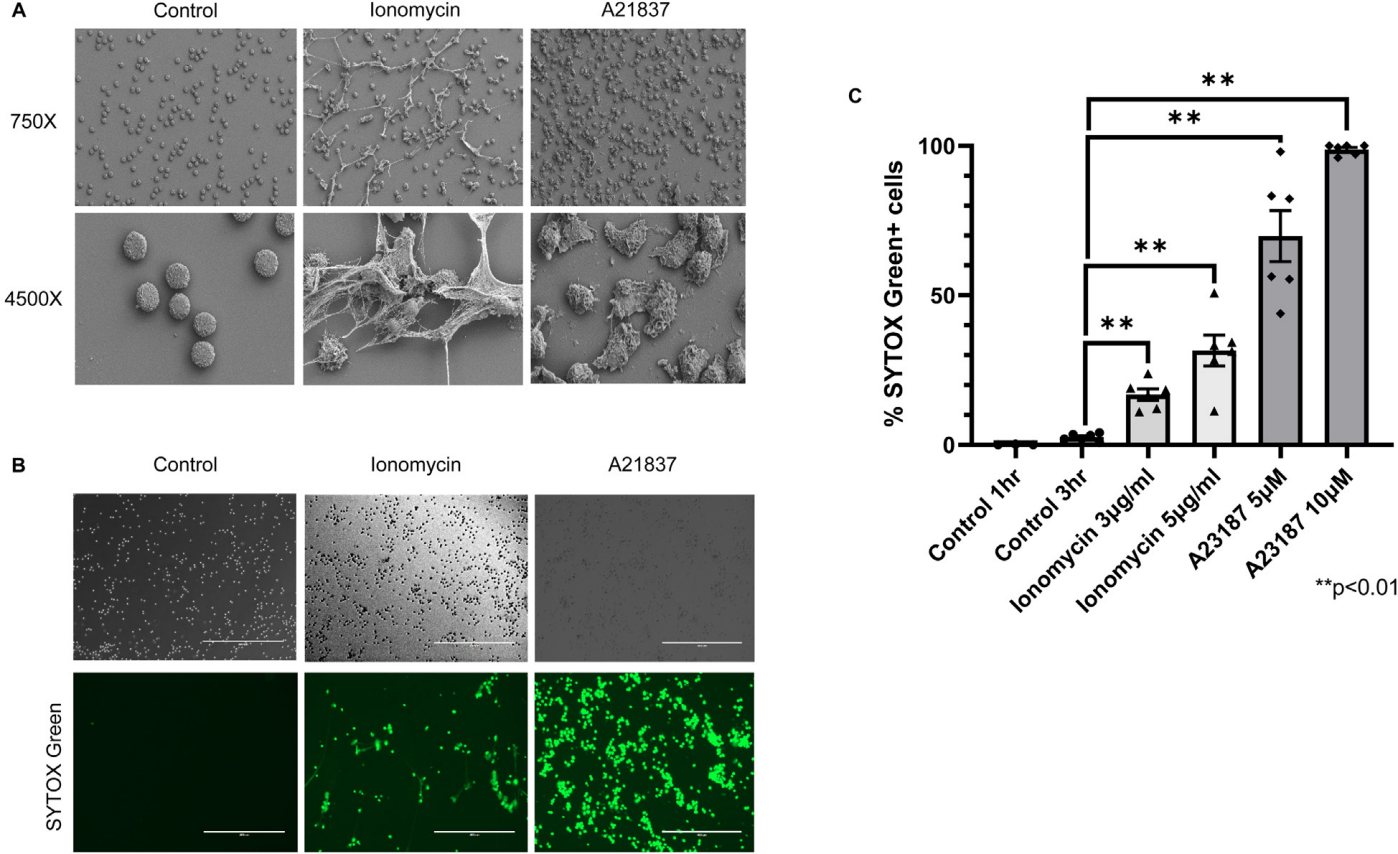
(A) Representative images of scanning electron microscopy of peripheral human neutrophils in control (media only) and treated with ionomycin (3μg/ml) and A23187 (5μM). (B) Representative images of neutrophils stimulated with ionomycin (3μg/ml) and A23187 (5μM) for 3 hours with SYTOX Green staining for DNA, magnification = 10×, scale bar = 400μm. (C). % SYTOX Green-labeled neutrophils over total neutrophil count following treatment with ionomycin and A23187; (neutrophils from one human donor, where each point represents a count from a well, in this case there were 6 counts taken (% SYTOX Green + neutrophils) from 3 wells (3 technical replicates) per treatment type. Data presented as mean + standard error of mean (SEM).

Using SEM, we first show that treatment of human peripheral blood neutrophils with 2 well-documented NET stimulants A23187 and ionomycin, both ionophores, resulted in marked morphological changes of the neutrophil with ionomycin leading to characteristic appearances of NET formation (Figure 1A). In these conditions, we treated the neutrophils with SYTOX Green, a dye that stains DNA and is impermeable to live cells. In the control condition of unstimulated neutrophils, there is no SYTOX Green staining whilst NET-stimulants A21387 and ionomycin resulted in a significant increase in the proportion of neutrophils with SYTOX Green labeling (Figure 1B). By enumerating SYTOX Green-labeled neutrophils/non-labeled neutrophils (presented in %), we showed that ionomycin and A23187 resulted in higher numbers of neutrophils labeled with SYTOX Green in a concentration-dependent manner (Figure 1C). On this basis, we use SYTOX Green staining as a marker for cell permeability and where visible, neutrophil DNA release in known experimental conditions that we will use for NET formation.

### GMCSF and LPS stimulation of human peripheral neutrophils does not result in NET formation

**Figure 2:**
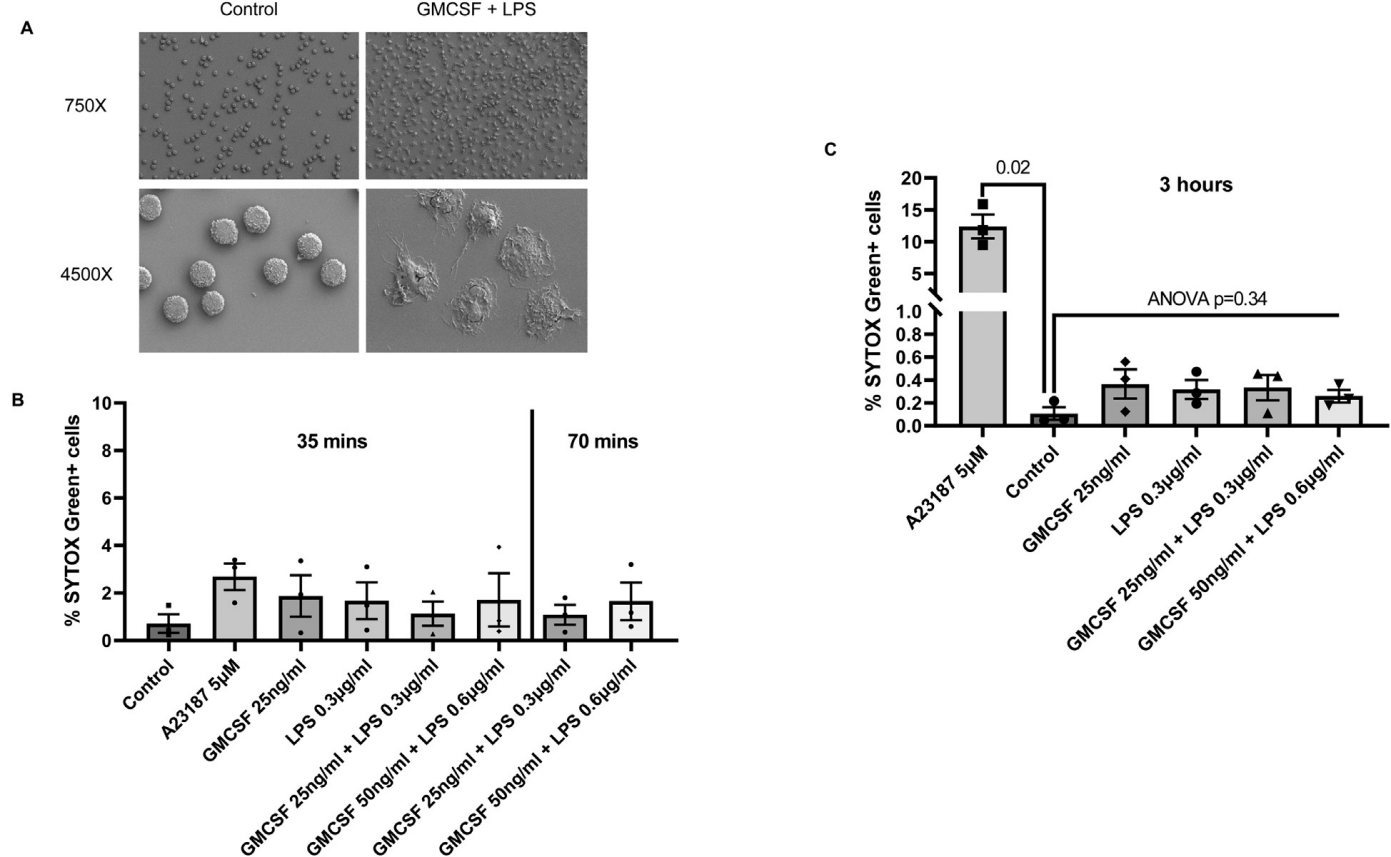
(A) Representative scanning electron microscopy images of peripheral human neutrophils following 3-hour treatment of GMCSF (25ng/ml) and LPS (0.3 μg/ml). (B) % SYTOX Green-labeled neutrophils over total neutrophil count following A23187, GMCSF, LPS, and GMCSF + LPS treatment (concentrations shown in the figure) over 35 and 70 minutes, respectively, (n = 3/group; n = 3 (neutrophils from 3 separate human donors were used, each point is an average of 6 counts (from 3 wells) per treatment group). (C) % SYTOX Green-labeled neutrophils over total neutrophil count following A23187, GMCSF, LPS, and GMCSF + LPS treatment (doses shown in the figure) over 3 hours; n = 3 (neutrophils from 3 separate human donors were used, each point is an average of 6 counts (from 3 wells) per treatment group. Two sets of different healthy human donors were used for experiments in Figure 2A and B. GMCSF, granulocyte macrophage colony-stimulating factor; LPS, lipopolysaccharide. Data presented as mean + standard error of mean (SEM).

We specifically investigated the effects of GMCSF and LPS, conditions previously described to induce mitochondrial NET formation.^9^ Here, GMCSF and LPS caused morphological changes characteristic of an activated neutrophil from a resting spherical shape to a flattened shape with directional pseudopods^10^ rather than NET formation (Figure 2A). We systematically screened our SEM images and did not observe any free extracellular mitochondria outwith neutrophils in both conditions. Yousefi et al. reported the selective release of mitochondrial (and not genomic) DNA by neutrophils when primed with GMCSF followed by LPS or C5a stimulation for a short period of 35 minutes.^9^ We tested similar short time points (35 and 70 minutes) with GMCSF and LPS and found no evidence of increased neutrophil DNA release (as measured by uptake of SYTOX Green by neutrophils) (Figure 2B). Even with treatment with known NET-inducer A23187, we observed only a small increase in neutrophil staining over these time periods (Figure 2B) which are very short stimulation periods for typical NET formation. As most known stimulants of NET formation have been shown to trigger NETs after 2–4 hours,^11^ we extended our stimulation period to 3 hours. As expected, A23187 treatment for 3 hours significantly increased SYTOX Green-labeled neutrophils (2.7% vs 12.0% in 35 minutes vs 3 hours, respectively, *P* = .02) (Figure 2C). At 3 hours, there were no differences in the frequencies of SYTOX Green-labeled neutrophils in GMCSF + LPS, GMCSF, and LPS-only treated groups compared to controls (analysis of variance *P* = .32 across these 5 experimental groups) (Figure 2C). The overall mean of GMCSF + LPS, GMCSF, and LPS-only treated groups are very low compared with the A23187 treatment group (0.4% vs 12%, respectively). Taken together, our data did not provide compelling evidence of NET formation through morphological changes or SYTOX staining under conditions previously described (GMCSF + LPS) to generate specific mitochondrial NET formation.

### GMCSF and LPS stimulation of human peripheral neutrophils leads to DNA release but lower mitochondrial DNA in comparison to A23187-induced NET formation

**Figure 3:**
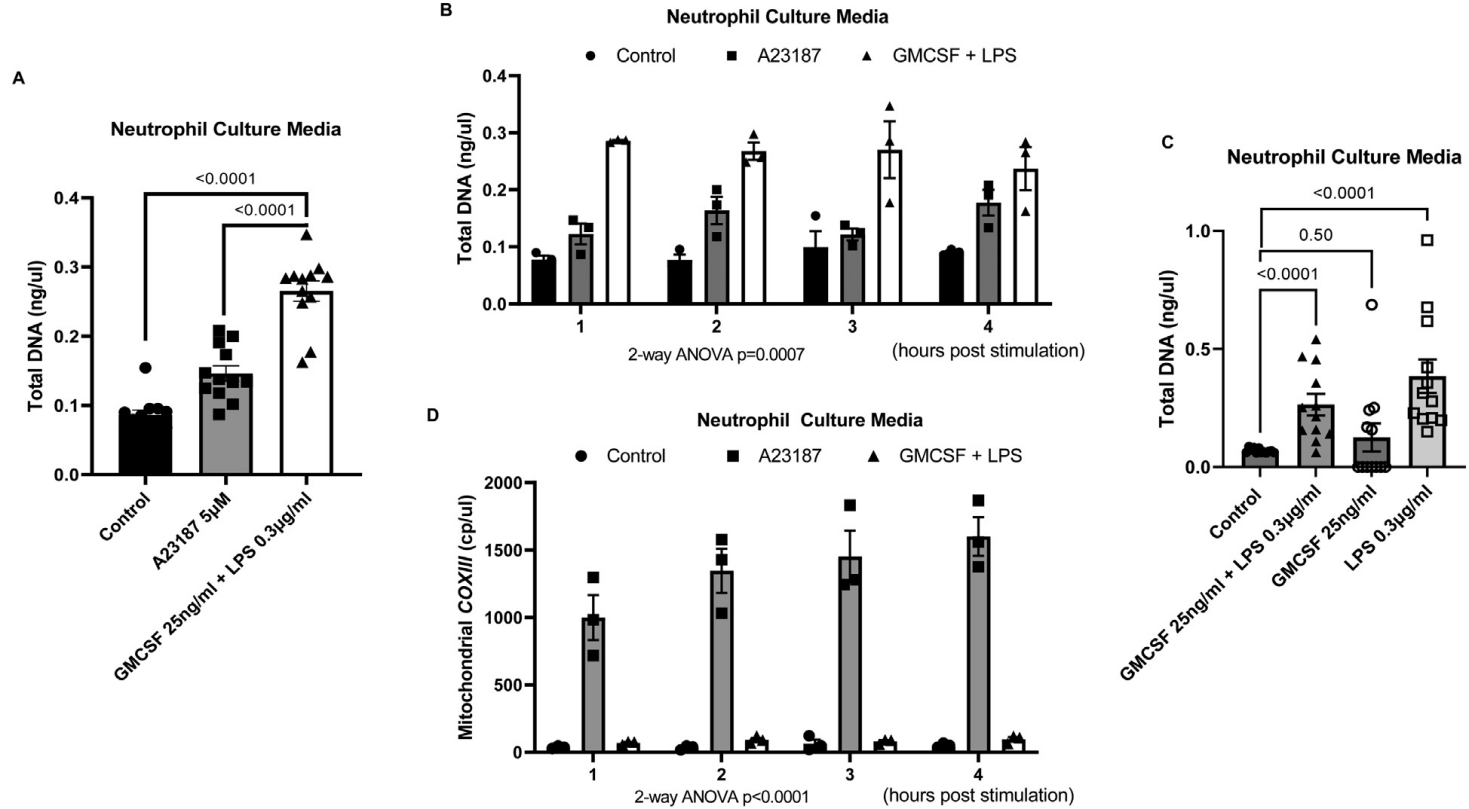

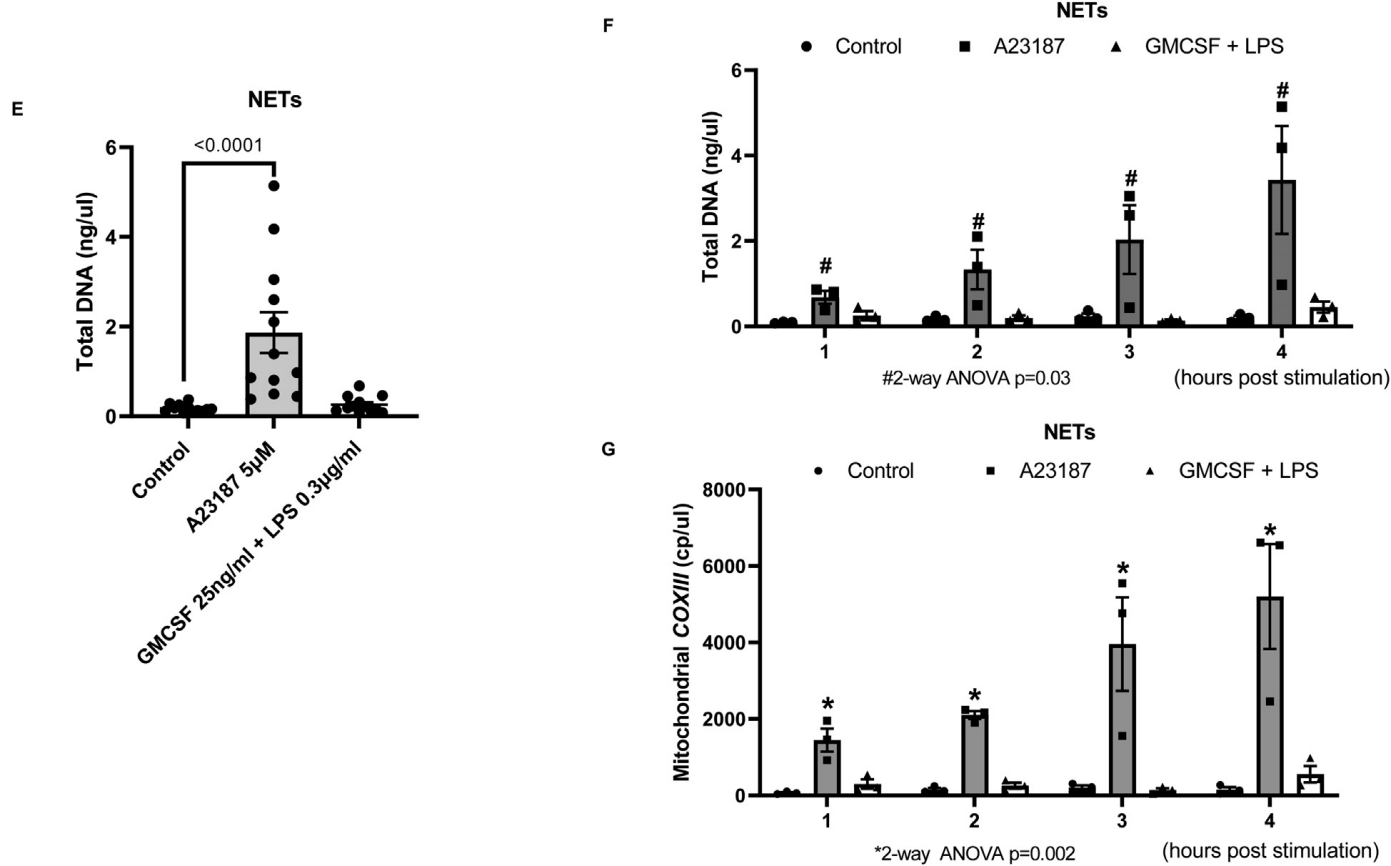
(A) Total DNA (ng/μl) measured from neutrophil culture medium following A23187 and GMCSF + LPS treatment‡. For each treatment group, 4 time points are combined for total of 12 data points/group). (B) Total DNA (ng/μl) measured from neutrophil culture medium following A23187 and GMCSF + LPS treatment over 4 hours†. (C) Total DNA (ng/μl) measured from neutrophil culture medium following GMCSF + LPS, GMCSF only and LPS only treatment‡. (D) Mitochondrial *COXIII* transcripts measured from neutrophil culture medium following A23187 and GMCSF + LPS treatment over 4 hours† using digital PCR. (E) Total DNA (ng/ul) measured from NETs PBS wash for well base following A23187 and GMCSF + LPS treatment‡. (F) Total DNA (ng/ul) measured from NETs PBS wash following A21387 and GMCSF + LPS treatment over 4 hours†. (G) Mitochondrial *COXIII* transcripts measured from NETs PBS wash following A23187 and GMCSF + LPS treatment over 4 hours (n = 3/group) using digital PCR. Two-way ANOVA tests used to compare control vs A23187 vs GMCSF + LPS. ‡Neutrophils from 3 separate human donors were used, each point is an average of 6 counts from 3 wells. For each treatment group, 4 time points are combined for total of 12 data points/group). †Neutrophils from 3 separate human donors were used, each point is an average of 6 counts from 3 wells. Data presented as mean + standard error of mean (SEM).

Although GMCSF and LPS stimulation of human peripheral neutrophils did not result in higher NET formation over short (35–70 minutes) and long (3 hours) periods, we further investigated if these stimuli can lead to extracellular DNA release. Following 4 hours of GMCSF and LPS treatment, we found significantly higher levels of total DNA in the cultured media of neutrophils as quantified in total DNA extracted using Qubit when compared to controls (*P* < .0001) (Figure 3A). Of interest, this is also significantly higher when compared with A23187 treatment that induces NET formation (*P* < .0001) (Figure 3A). Longitudinal analysis, over hourly time points, showed that DNA release occurred early within the first hour of stimulation, with no further increase after this (Figure 3B). We compared the relative effects of GMCSF and LPS and found that it is the latter that drives the observation of neutrophil DNA release. Here, LPS only resulted in significantly higher DNA release which is not observed in GMCSF-only group (*P* < .0001 and *P* = .50, respectively) (Figure 3C). We subsequently investigated the GMCSF and LPS compared to the condition of NET formation (here, the A23187 group) that is specifically associated with mitochondrial DNA release, using mitochondrial-specific *COXIII* gene digital polymerase chain reaction (PCR) of extracted DNA from cultured media. In both A23187 and GMCSF + LPS groups, mitochondrial-specific *COXIII* gene transcripts were significantly higher in the cultured media compared to untreated controls (analysis of variance *P* < .0001) (Figure 3D). Of interest, however, there is a 20-fold higher increase in mitochondrial *COXIII* copy number in the A23187 treatment group (Figure 3D). This suggests that the DNA released by neutrophils treated with A23187 is proportionately far higher in mitochondrial reads despite there being less overall DNA released than GMCSF + LPS-treated neutrophils. Hence, our data show that NET formation is associated with higher mitochondrial DNA release.

### A23187-induced NETs contain high levels of mitochondrial DNA

We next used a different method to investigate whether higher mitochondrial DNA release found with A23187-NET formation can be attributed to mitochondrial DNA bound to NETs. Here, following the removal of media from the cultured neutrophils, we undertook washings using PBS of the well bases containing adherent NETs. DNA was then extracted from the PBS wash thereby providing a closer DNA measurement of NET content. A23187 treatment resulted in higher total DNA and mitochondrial DNA levels compared with GMCSF and LPS and controls (Figure 3E and F). In addition, the absolute levels of total and mitochondrial DNA in this approach are higher (approximately 10-fold) in PBS wash supernatants compared with that in the cultured media (Figure 3A, C and E). We postulate that higher levels of DNA adhere to the well base during in vitro NET formation under A23187 treatment leading to lower free-circulating DNA as seen in Figure 3A. Furthermore, the in vitro conditions of GMCSF + LPS treatment may lead to free-circulating DNA release that is independent of NET formation but present at lower levels. Using this approach, total DNA and mitochondrial DNA (*COXIII* transcripts) were significantly higher in PBS washing of adherent NETs following A23187 treatment compared to control and GMCSF + LPS treatment. (Figure 3F and G). We were also able to reliably quantify increasing levels of genomic DNA using GAPDH-targeted dPCR over time under A23187 treatment (data not shown).

### GMCSF + LPS stimulation and A23187-induced NETs showed different patterns of DNA fragment release

**Figure 4:**
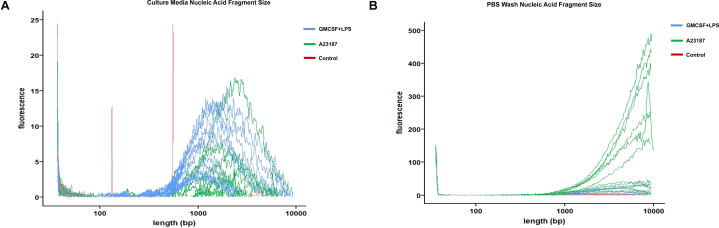

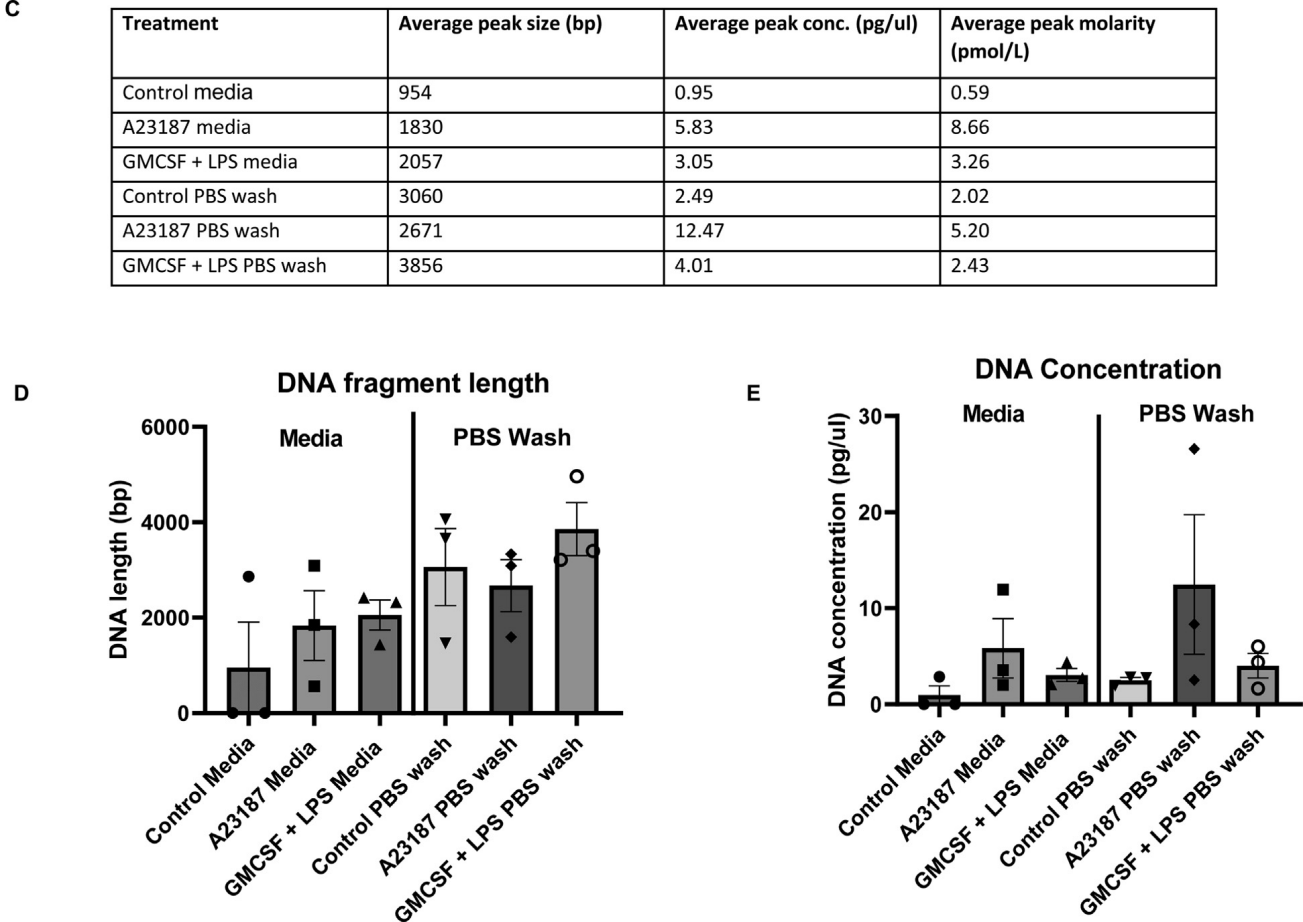
(A) DNA fragment length analysis using Bioanalyzer neutrophil culture medium following A21387 (Green), GMCSF + LPS (Blue), and control (Brown) treatment over 4 hours†. (B) DNA fragment length analysis using Bioanalyzer in PBS NETs wash following A21387 (Green), GMCSF + LPS (Blue), and control (Brown) treatment over 4 hours†. (C) Summary of DNA peak lengths, concentrations and molarity neutrophil culture medium and PBS NETs wash following A23187 and GMCSF + LPS treatment over 4 hours†. Media indicates where media was taken off stimulated neutrophils and a supernatant was prepared, and PBS wash indicates where PBS was used to wash the well base and a supernatant was prepared. DNA was extracted from these supernatants and measured with a bioanalyzer. (D) DNA fragment length of neutrophil culture medium and PBS wash conditions^a^. (E) DNA concentration of neutrophil culture medium and PBS wash conditions^a^. †For each treatment groups, 3 separate human donors were used with 4 hourly time points (1,2,3, and 4 hours). Data are presented for each time point, giving 12 data points/per treatment group. ^a^ Each data point is a mean of 4 time points (1, 2, 3, and 4 hours) for one human donor. **NB.** For Figure 4A and B red control lines not visible due to very low concentrations of released DNA fragments.

We further investigated the 2 different conditions, GMCSF + LPS and A23187-induced NET formation to determine whether there are qualitative differences of the pattern of DNA release. Recent studies suggest that DNA fragment lengths are critical to the type of nucleic acid sensor activation and therefore inflammatory responses generated.^12^ Here we used fragment analysis of DNA in the 2 experimental conditions described above – (a) in the neutrophil culture medium; and (b) in the PBS wash of well bases following removal of medium (Figure 4A and B, respectively). The overall pattern of DNA fragment release was different between these 2 conditions (Figure 4A and B). In the latter condition that is more reflective of adherent NETs to well bases, higher quantities of long DNA fragment lengths (>10, 000 base pairs) were observed, particularly in A23187-induced NETs (Figure 4B). In both these conditions, however, there were no significant differences in average peak size of DNA lengths between A23187 and GMCSF + LPS treatments (Figure 4D), this analysis, however, is limited by the methodology with a maximum cutoff value of 10, 000 base pairs. The concentrations of DNA release following A23187-induced NETs were higher but not statistically significant in both neutrophil culture medium and PBS wash (Figure 4E).

### Heterologous plasma coculture from patients with active IBD do not result in significantly higher mitochondrial DNA release or NET formation

**Figure 5:**
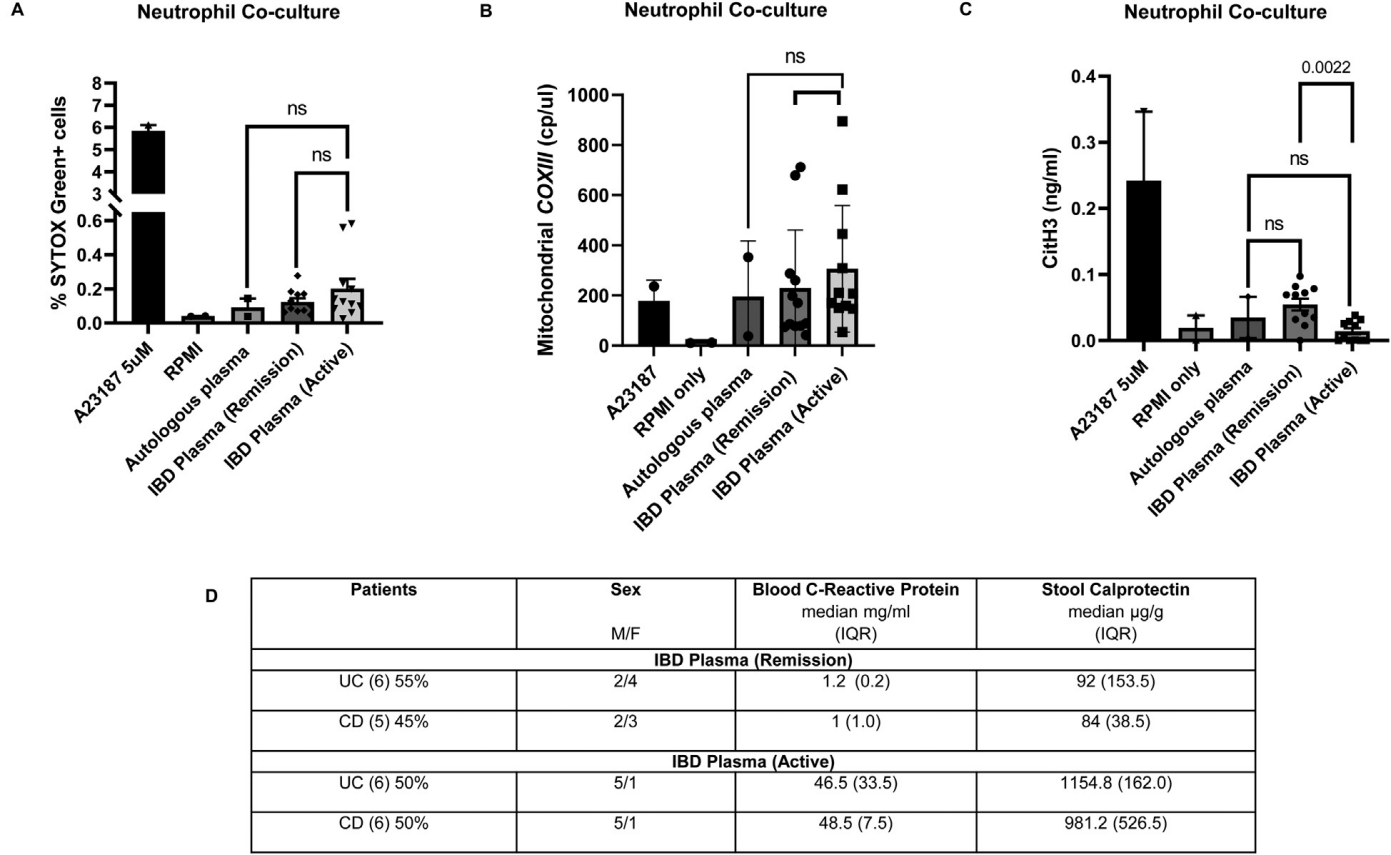
(A) % SYTOX Green-labeled neutrophils over total neutrophil count following human peripheral neutrophil coculture with A21387, RPMI media only, coculture with autologous plasma, heterologous IBD plasma from patients in remission and with active disease (n = 2, 12 and 12, respectively). (B) Mitochondrial *COXIII* transcripts measured from media supernatants following human peripheral neutrophil coculture with A23187, RPMI media only, coculture with autologous plasma, heterologous IBD plasma from patients in remission and with active disease (n = 2, 12 and 12, respectively). (C) CitH3 ELISA measured from media supernatants following human peripheral neutrophil coculture with A21387, RPMI media only, coculture with autologous plasma, heterologous IBD plasma from patients in remission and with active disease (12 and 12, respectively). (D) Patient details for IBD samples used in coculture experiments carried out in this section. Data presented as mean + standard error of mean (SEM). IBD, inflammatory bowel disease; UC, ulcerative colitis; CD, Crohn’s disease; CitH3, citrullinated histone H3.

Given that both conditions, GMCSF + LPS and A23187-NET induction of peripheral blood neutrophils were associated with higher total and mitochondrial DNA in vitro, we tested if plasma from our patients with highly active IBD can trigger a similar process. We posit that there are potential factors in the blood that can promote NET formation or DNA release from neutrophils during an active flare of IBD. Here, healthy donor neutrophils from one donor were cultured in autologous plasma; and plasma from 12 IBD patients with active disease (6 UC/6 CD; blood C-reactive protein median 48 mg/ml; stool calprotectin median 1067 μg/g) and 12 patients in clinical remission (blood C-reactive protein median 1 mg/ml; stool calprotectin median 95 μg/g) for 4 hours (Figure 5D). Firstly, we did not find increased NET formation (as evidenced in % of SYTOX Green labeled neutrophils and CitH3 levels that are released during NET formation) in neutrophils cultured in heterologous plasma from IBD patients with active disease and in remission compared with neutrophils cocultured in autologous plasma (Figure 5A and C). Neutrophils undergo hypercitrullination of histone H3 by peptidylarginine deiminase 4 leading chromatin decondensation during NETosis.^13^ Secondly, although there was a trend toward higher mitochondrial DNA (mitochondrial *COXIII* copy numbers) released into media when neutrophils were treated with plasma from highly active vs clinical remission groups, this difference was not statistically significant (median 207 vs 129 copies/μl, respectively, *P* = .3) (Figure 5B). Of interest and unexpectedly, CitH3 levels were higher following heterologous culture with plasma from patients in remission compared with those from active disease (*P* = .002) (Figure 5C). It is noteworthy however; that the overall range of citH3 levels at the low range of values that can be detectable by the ELISA kit.

### CitH3 ELISA levels are lower in patients with active IBD

**Figure 6:**
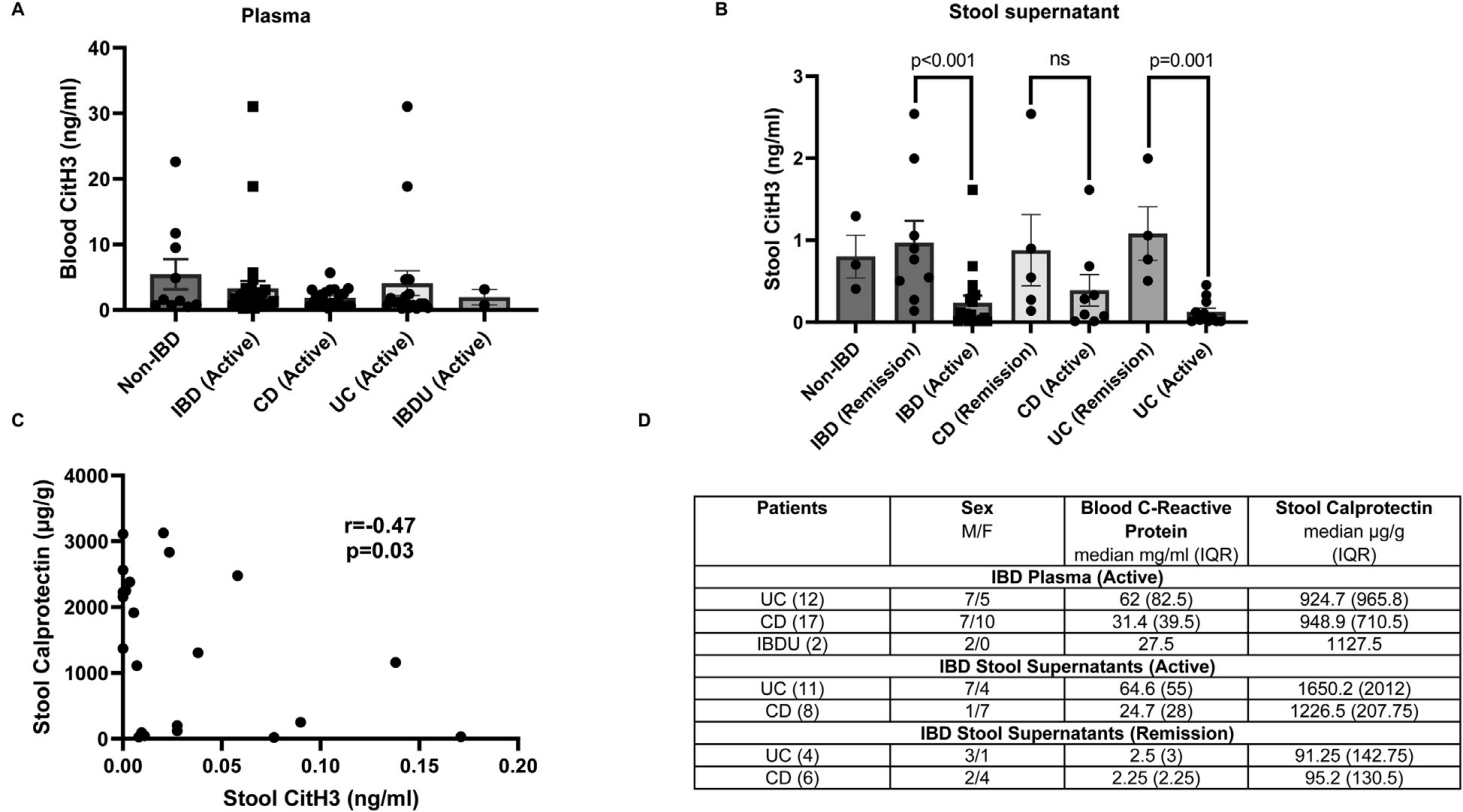
(A) CitH3 ELISA measured from plasma of non-IBD and IBD (UC and CD subsets) patients with active disease (n = 10 and 31, respectively). (B) CitH3 ELISA measured from stool supernatants of IBD patients (with UC and CD subsets) in remission and with active disease (n = 10 and 19, respectively). (C) Spearman correlation analysis between stool calprotectin and CitH3 (n = 22-paired observations). (D) Patient details for IBD samples used in coculture experiments carried out in this section. Data presented as mean + standard error of mean (SEM).

Critically ill patients demonstrate excessive NET formation in the blood in conditions such as severe Covid19 and sepsis; a process that contributes directly to immune-pathology and clinical mortality.^14^^,^^15^ In such cases, circulating NETs can be detected in blood by the presence of NET-specific markers such as CitH3, which has been demonstrated as a NET marker out with phorbol 12-myristate 13-acetate-driven NET formation.^16^, ^17^, ^18^ We tested plasma from 31 patients with highly active IBD vs 10 non-IBD controls; but found no evidence of higher CitH3 blood levels in IBD patients with an active flare vs non-IBD patients (Figure 6A). As IBD inflammation is localized to the gut (vs a systemic process), we further tested CitH3 in stool supernatants of IBD patients with 19 highly active IBD vs 10 remission IBD. We found very low levels of CitH3 despite high levels of stool calprotectin (median of 1440 μg/g in active IBD supernatants [>100 μg/g is considered clinically elevated]), a neutrophil-linked IBD biomarker. Paradoxically, we observed significantly higher levels of CitH3 in stool supernatants of IBD patients in remission compared to those with an active flare of disease (*P* < .001); a pattern which is observed in both UC and CD respectively (Figure 6B). There is a negative correlation between stool calprotectin and CitH3 (r = 0.47, *P* = .03) (Figure 6C). Hence, using CitH3 as a surrogate marker for circulating NETs, our data suggest that excessive NET formation is unlikely to play a dominant role in blood DNA (and mitochondrial) release in IBD.

## Discussion

Our current study investigates the importance of neutrophils in the release of circulating mitochondrial DNA in IBD. Here, we focused on the roles of NET formation and the described, specific mitochondrial NETosis induced by in vitro conditions of GMCSF and LPS stimulation (previously reported by Yousefi *et al.*) as potential contributory processes. We then sought further evidence for these 2 factors in our patients with active IBD. Our data show that NET formation induced by A23187 ionophore resulted in higher total DNA and mitochondrial DNA release with a tendency towards longer fragments of DNA. This is unsurprising, given the known nature of NETs where long scaffolds of DNA and histones are released during the process of NETosis. In relation to mitochondrial NETs, we could not show specific mitochondrial NET formation or expulsion of mitochondria using our methodology, a process described Yousefi *et al.*; and we found no evidence of NET-formation following GMCSF and LPS treatment of human neutrophils. Of interest, our data show that GMCSF and LPS (typically leading to neutrophil activation), resulted in higher DNA release in neutrophils without NET formation. A23187-induced NET formation resulted in higher mitochondrial DNA release compared to GMCSF and LPS treatment. Therefore, the latter process may play a lesser role in neutrophil mitochondrial DNA release quantitatively. From a physiological perspective, this restrained capacity to release highly pro-inflammatory mitochondria DNA during activated neutrophilic states is rational to avoid excessive amplification of inflammation.^19^ This is in contrast with NET formation, where large amounts of sticky DNA need to be released as a physical trap or web to neutralize invading pathogens for example.

Given that, these in vitro conditions do not capture IBD pathophysiology, we explored the possibility that plasma from patients active IBD may promote NET formation and trigger mitochondrial DNA release from neutrophils to provide contextual relevance to human disease. Our data using heterologous coculture with IBD plasma did not show this. We also sought evidence for excessive NET formation in active IBD by measuring a NET marker, CitH3. As indicated earlier, high levels of circulating NETs in blood are associated with conditions of overwhelming systemic physiologic compromise, such as severe COVID-19 where NETs also contribute to collateral tissue damage and other pathogenic developments such as thrombosis.^14^^,^^18^^,^^20^ In blood, we did not find elevated CitH3 levels to indicate excessive NET formation in active IBD. In stools, CitH3 levels were significantly lower in active IBD, which is paradoxical. There may be post-translational modifications of CitH3 and/or inhibitory factors in the active IBD stool supernatants that could potentially influence and confound the CitH3 ELISA test. More likely, high levels of proteases in the active IBD gut can degrade NETs in our stool supernatants, unlike calprotectin, which is stable and usually resistant to breakdown.^21^

In the context of human disease, IBD patients do not have the magnitude of tissue damage seen in critically unwell patients, for example, with Systemic inflammatory response syndrome that can lead to widespread activation of NET formation to levels where NETs can be detected in blood.^4^^,^^15^^,^^20^ Notwithstanding this, NETosis is a crucial gut defense mechanism and several lines of evidence show that excessive NET formation (or inadequate clearance) can perpetuate the gut inflammatory response in IBD.^22^, ^23^, ^24^, ^25^ While our data suggest that NET formation in blood neutrophils is unlikely to be a main contributor to high circulating mitochondrial DNA in IBD, the importance of activated neutrophils in this regard is unclear. Our data (and others) show that in vitro stimuli like the *bona fide* PAMP such as LPS can induce DNA release without NET formation. Caielli *et al* showed that live health neutrophils can extrude mitochondrial DNA that is complexed to mitochondrial matrix proteins in the absence of stimulation.^26^

There are limitations to our study. Firstly, we did not test blood neutrophils from patients with active IBD. Our ethics approval did not permit the volume of blood required for such experiments in our patients. Secondly, our patient group sizes, in particular, heterologous coculture experiments were small. More expansive screening of neutrophils (and their phenotypes) in our IBD (with UC and CD) populations could be feasible, if the initial data were supportive. Thirdly, we do not show the mechanism for GMCSF and LPS-mediated in vitro mitochondrial DNA release. Finally, NET formation is a dynamic process with tissue-specific factors. Hence, our range of NET-assays is not broad enough to capture NET formation in IBD. In particular, SYTOX Green staining of neutrophils is not definitive evidence of NET formation or DNA release as such but rather a sign of an increased permeability in the cells. The morphology of NETs under differing stimulants is under debate and both fibrous and ‘cloud-like’ NETs are still accepted, thus defining what is and what is not a NET using SYTOX Green staining can be arbitrary.^16^ Here, we used SYTOX Green staining to show an increase in cell permeability and DNA release under classical NET stimulants which was absent in GMCSF + LPS-treated cells.

## Conclusion

Our data do not support excessive NET formation and mitochondrial NETs in active IBD as a potential factor for our previously observed high mitochondrial cell-free blood DNA levels.^1^ The importance of neutrophil activation leading to mitochondrial DNA release that is independent of NETosis and/or neutrophil inflammatory cell death in IBD requires more work, particularly with increasing data pointing towards the importance of the neutrophils as a pivotal module in the complex inflammatory landscape and treatment failure in IBD.^27^^,^^28^

## Materials and Methods

### IBD and control samples

Healthy donors were recruited from the Centre for Inflammation Research Blood Donor Register under the provision of Ethics Reference 21-EMREC-041. Plasma and stool samples were obtained from IBD patients from the Gastrointestinal Unit, Western General Hospital, Edinburgh (Ethics Reference 18/ES/0090). At the time of recruitment, clinical and biological data of IBD disease activity were recorded.

### Neutrophil isolation

Whole blood was collected in 3.8% sodium citrate and centrifuged at 350 x g for 20 minutes within 30 minutes of collection. Plasma was removed and polymorphonuclear cells were isolated by 6% dextran sedimentation and separation by discontinuous Percoll gradient (GE Healthcare, Buckinghamshire) at 81%, 68%, and 55%. Isolated cells were washed once with cation-free Dulbecco’s phosphate-buffered saline (DPBS−/−).

### DNA release assay

Neutrophils were plated at 200,000 or 400,000 cells per well in a 24-well plate in Roswell Park Memorial Institute (RPMI) media + 10% fetal calf serum (FCS) + 1% penicillin and streptomycin with 1% L-glutamine. They were plated either in RPMI only or RPMI with 5uM A23187, granulocyte macrophage colony-stimulating factor (Peprotech, London), LPS solution from *Escherichia coli* 026:B6 (Invitrogen, Inchinnan) or granulocyte macrophage stimulating factor (GMCSF) + LPS at various concentrations and timescales. Media was then stained with 0.15uM SYTOX Green dye (Life Technologies, Paisley) and imaged using an EVOS FL Basic in both bright field and green fluorescent protein (GFP) channels. Neutrophil images were converted on Fiji by the following methodology. Firstly, scale set with scale bar. Process > Subtract Background; Image > Type>8-bit; Image > Adjust > Threshold – threshold adjusted manually for each image to achieve greatest contrast, thereby isolating cells. Process > Binary > Convert to mask; Process > Binary > Fill holes; Process > Binary > Watershed; Analyse > Analyse particles > Size = 20–200μm2 > Circularity 0-1. GFP positive cells counted using DNA and NETosis Analysis (DANA) plugin.

### Quantification of overall DNA in NET supernatant

Neutrophils were cultured in RPMI + 10% FCS + 1% penicillin and streptomycin with 1% L-glutamine at 3 × 10^6^ cells per well in a 12-well plate. Neutrophils were stimulated with either 5 μM A23187, 25 ng/ml GMCSF, 0.3 μg/ml LPS, or GMCSF + LPS for 4 hours. At every hour, media was collected and 2ml PBS was used to wash each well base 20 times to isolate NET DNA and proteins (PBS wash). Media and ‘PBS wash’ was centrifuged at 450 × g for 10 minutes at room temperature. Supernatant DNA was extracted using the QIAamp Circulating Nucleic Acid kit (Qiagen, Manchester) with the Qiagen Vacuum Pump and QIAvac 24 plus vacuum manifold. DNA was quantified on a Qubit 4 fluorometer using the Qubit dsDNA High Sensitivity (HS) assay kit (Life Technologies, Paisley).

### Quantification of mitochondrial and genomic DNA in NET supernatant

The Stilla Naica Digital PCR (dPCR) system (Stilla Technologies, Villejuif) was used to quantify mitochondrial genes COX3 (FWR = 5′ CGAGTCTCCCTTCACCATTTC 3’; REV = 5′ TTGGCGGATGAAGCAGATAG 3’; probe = /56-FAM/CG ACG GCA T/ZEN/C TAC GGC TCA ACA TT/3IABkFQ/ - IDT, Coralville, Iowa) and ND2-HEX (FWR = 5′ ACCTGAGTAGGCCTAGAAATAAAC 3’; REV = 5′ GTTGCTTGCGTGAGGAAATAC 3’; probe = /5HEX/TC GTT CCA C/ZEN/A GAA GCT GCC ATC AA/3IABkFQ/ - IDT) as well as nuclear gene GAPDH-FAM, TaqMan Copy Number Assay, Assay ID: Hs00483111_cn, (Life Technologies, Paisley). All DNA was subject to EcoRI restriction enzyme (Life Technologies) digestion for 30 minutes at 37 °C prior to dPCR. Stilla Naica Sapphire chips (Stilla Technologies) were used, and the cycling parameters were as follows: initial heating to 95 °C for 3 minutes followed by 47 cycles of 95 °C for 15 seconds and 52 °C for 20 seconds. Chips were read on a Stilla Naica Prism 6 fluorescence imager (Stilla Technologies) with blue channel exposure set to 65ms and green channel set to 200 ms.

### NET DNA fragment analysis

DNA isolated from NET culture media or PBS wash was analzsed for concentration and fragment length using an Agilent 2100 Bioanalyzer with AGILDNA HS chips. Data were analyzed using an R-based online vignette ‘bioanalyzeR’ (Joe W Foley, bioanalyzeR, (2022), Github repository, https://github.com/jwfoley/bioanalyzeR).

### Quantification of plasma, stool and NET supernatant citrullinated histone 3

Citrullinated histone 3 (CitH3) ELISA kit (Cambridge Biosciences, Cambridge) was used to quantify plasma citH3. Plasma from healthy donors and IBD patients was collected by centrifuging whole blood, collected in an ethylenediaminetetraacetic acid tube for 10 minutes at 1000 × g and then again for 10 minutes at 3000 × g at RT before being frozen within 4 hours of collection. Plasma was defrosted and diluted 1:2 in assay buffer as stated by the manufacturer. NET supernatant was also diluted 1:2 with assay buffer. Stool samples were defrosted, diluted 1:50 with extraction buffer using an Easy Extract device (Firefly Scientific, Worsley) and vortexed for 3 minutes to create stool supernatant. Supernatant was frozen at −80 °C until needed. Stool supernatant was then diluted 1:2 with assay buffer from the same CitH3 ELISA kit and CitH3 was quantified as instructed by the manufacturer.

### Coculture of healthy donor neutrophils with plasma from IBD patients

Neutrophils were isolated from one healthy donor and cultured for 4 hours in a 1:1 mix of RPMI media + 10% FCS + 1% penicillin and streptomycin with 1% L-glutamine and IBD patient plasma, autologous plasma or 5uM A23187 (in RPMI). Plasma was collected in an ethylenediaminetetraacetic acid tube and centrifuged for 10 minutes at 1000 × g and then again for 10 minutes at 3000 × g at RT before being frozen within 4 hours of collection. After 4 hours the culture media was centrifuged for 10 minutes at 450 × g and the supernatant was used to measure CitH3 using the same ELISA as in other experiments, total DNA and mitochondrial DNA. Culture supernatant was frozen at −20 °C until needed.

### Scanning electron microscopy

Neutrophils were isolated from whole blood from healthy donors as above and incubated for 3 hours in RPMI + 10% FCS + 1% penicillin and streptomycin with 1% L-glutamine. Neutrophils were either untreated (control) or treated with A23187 (5μM), ionomycin (5ug/ml) or GMCSF (50ng/ml) + LPS (0.6ug/ml). Media was removed and cells were fixed in 3% glutaraldehyde **(…)** in 0.1M sodium cacodylate buffer, pH 7.3 **(…)** for 2 hours. Cells were then gently washed 3 times for 10 minutes each in 0.1M sodium cacodylate buffer. Cells were left in 0.1M sodium cacodylate buffer overnight. Sodium cacodylate was removed and 500μl PBS was added to the cells. Samples were then postfixed in 1% osmium tetroxide in 0.1M sodium cacodylate buffer for 45 minutes. Three further 10-minute washes were performed in 0.1M sodium cacodylate buffer. Cells were then dehydrated in graded concentrations of acetone (50%, 70%, 90% and 3 times in 100%) for 10 minutes each. Cells were then dried by critical point drying using liquid carbon dioxide. Samples were then mounted on aluminum stubs and sputter coated in gold palladium before analysis on a Zeiss Eigme HD VP scanning electron microscopy (SEM).

### Statistics

All statistical analysis was carried out on R or GraphPad9. For parametric and nonparametric data, t-test and Mann-Whitney tests were used, respectively, with a two-tailed *P*-value of < .05 as significant. For comparisons of 3 groups, a 2-way ANOVA test with Geisser-Greenhouse correction was used. Variability and normality were assessed using the Fligner-Killeen test and the Shapiro-Wilk test, respectively, for each data set prior to statistical tests.
